# The potential impact of habitual sleep quality on glycaemic control and inflammation: A study on geriatric patients recently diagnosed with type 2 diabetes mellitus (T2DM)

**DOI:** 10.1016/j.sleepx.2025.100139

**Published:** 2025-03-07

**Authors:** Nadia Hussain, Amal Hussain Ibrahim Al Haddad, Saima Abbass, Zina Alfahl

**Affiliations:** a Department of Pharmaceutical Sciences, College of Pharmacy, Al Ain University, United Arab Emirates; b AAU Health and Biomedical Research Center, Al Ain University, Abu Dhabi, United Arab Emirates; c Chief Operations Office, Sheikh Shakhbout Medical City (SSMC), PureHealth, Abu Dhabi, United Arab Emirates; d Department of Internal Medicine, Shifa Hospital, Lahore, Pakistan; e Antimicrobial Resistance & Microbial Ecology Group, School of Medicine, University of Galway, Galway, Ireland; f Centre for One Health, Ryan Institute, University of Galway, Galway, Ireland

**Keywords:** Diabetes mellitus, Type 2 diabetes mellitus, Sleep quality, Geriatric, Inflammation

## Abstract

Sleep quality and its relationship with glycaemic control is of particular interest in the context of geriatric diabetes. We aimed to investigate the potential impact of habitual sleep quality on glycaemic control status among geriatric patients recently diagnosed with type 2 diabetes mellitus (T2DM). A total of 193 geriatric patients recently diagnosed with T2DM in a tertiary-care hospital were selected. A developed questionnaire was used to assess various aspects of sleep quality. Glycaemic control was evaluated through fasting blood glucose levels, HbA1c measurements and number of admissions to the hospital for hypoglycaemic or hyperglycaemic episodes. Patients were divided into Poor Sleep Quality (PSQ, n = 132) and Adequate Sleep Quality (ASQ, n = 61) groups. The PSQ group exhibited significantly worse sleep outcomes, including longer sleep latency (35 ± 9.2 min vs. 15 ± 6.4 min), shorter sleep duration (5 h 42 min vs. 7 h 18 min) and greater use of sleep medications (72 % vs. 22 %). Glycaemic control, measured by HbA1c, was worse in the PSQ group (8.7 ± 1.9 vs. 7.2 ± 1.2; p < 0.01), which also had more frequent severe hypoglycaemic (35 ± 1.4 vs. 8 ± 2.1; p = 0.02) and ketoacidotic episodes (72 ± 1.0 vs. 5 ± 1.1; p = 0.01). These findings suggest an association between poor sleep quality and poorer glycaemic control, with more frequent diabetes-related complications, highlighting the need for further research to explore potential causal relationships and targeted interventions in this population.

## Highlights

• Older adults with Type 2 diabetes face challenges in glucose regulation.
• Poor sleep quality impacts glucose control, inflammation, and health outcomes.
• 193 participants categorized into poor vs. adequate sleep quality groups.
• Poor sleep group had higher blood glucose and more hospitalizations.
• Improving sleep quality may enhance diabetes management and overall health.

## 1 Introduction

**Fig. 1:**
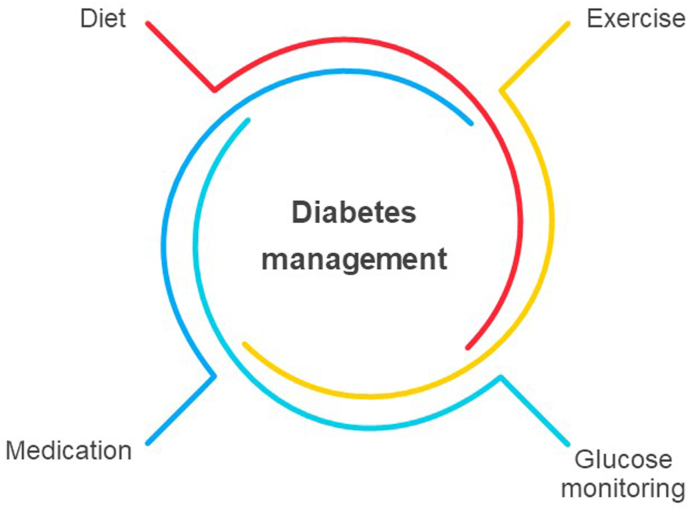
Diabetes management strategies.

Diabetes mellitus (DM) is a chronic disease characterized by impaired insulin secretion, insulin resistance or both which leads to a host of devastating complications [1]. Maintaining adequate glycaemic control remains the cornerstone for reducing the risk and severity of diabetic complications and minimizing the impact on patients' lives [2]. This requires the persistent management of diabetes via diet, exercise, medication and glucose monitoring (Fig. 1).

Type 2 diabetes mellitus (T2DM) is a major public health concern, particularly in low- and middle-income countries, which account for approximately 75 % of the global burden of diabetes [1]. Pakistan has one of the highest prevalence rates of diabetes in the world, with an estimated 26.7 % of adults (approximately 33 million people) affected in 2022, making it the country with the highest adult diabetes prevalence globally [1]. The growing burden of T2DM is particularly concerning among the geriatric population, who are at higher risk of complications.

Emerging evidence suggests that sleep disturbances may influence glycaemic control in individuals with T2DM. Several studies have explored the relationship between sleep quality, glycaemic control and diabetes-related complications [2,3], but data from Pakistan, where diabetes prevalence is highest, remains scarce. Our study aims to fill this gap by evaluating the association between sleep quality and glycaemic control in geriatric patients recently diagnosed with T2DM. Understanding this relationship in a high-burden population may inform targeted interventions to improve diabetes management.

T2DM imposes a considerable burden on the population's health and has many modifiable and non-modifiable risk factors associated with it [4,5]. Sleep is a vital process that has an impact on glucose metabolism in T2DM individuals [6]. Individuals with T2DM appear to be more susceptible to sleep issues with prevalence rates being up to 76.8 % [7]. The combination of diabetes and sleep issues poses a greater threat to an individual's health [8]. Issues with sleep has also been linked to the development of T2DM [9]. Sleep-related issues encompass problems with both the initiation and maintenance of sleep, while sleep disorders meet specific diagnostic criteria and require precise classification. This study focused on sleep quality, as individuals can encounter these problems at any stage of life, regardless of their medical history [7]. Timely and appropriate interventions could help alleviate sleep disturbance in people with diabetes. Recent recommendations by the American Diabetes association emphasized that sleep assessments should be included in all medical evaluations of patients with diabetes [10].

The aim of this study was to investigate the potential impact of habitual sleep quality on glycaemic control status among Pakistani geriatric patients recently diagnosed T2DM, specifically on HbA1c and the number of hypoglycemic or hyperglycaemic episodes. Specifically, we assessed the HbA1c values, fasting blood glucose and frequency of hypoglycaemic or hyperglycaemic episodes in this population. We hypothesized that those with poor quality of sleep had inadequate glycaemic control indicated by higher HbA1c levels and increased number of hospital admissions linked to hypoglycaemic or hyperglycaemic conditions.

## 2 Materials and methods

### 2.1 Study design and ethical approval

This study is a cross-sectional; single-centre study conducted at Shifa Hospital, Pakistan. The study was conducted in agreement with the ethical guidelines of the Declaration of Helsinki, Good Clinical Practice guidelines and all applicable regulatory requirements. All enrolled patients provided a written informed consent to participate after being informed about the study's purpose, potential risks and benefits and the data collected. Informed consent was obtained in accordance with the guidelines of the Shifa Hospital Board Institutional Review Board (Ethical approval no. ESH-34987355). Participants were assured that their data would be kept confidential and anonymized.

### 2.2 Study participants

The study included both male and female patients with T2DM who were enrolled and followed up through the clinical database. The research utilized a source population of patients registered between May 1, 2023 and October 1, 2023 at Shifa Hospital, Pakistan. From the registry, 228 potential participants were identified and contacted, with those declining to participate excluded from the study.

The inclusion criteria were male and female geriatric patients recently diagnosed with T2DM within the last 24 months and patients over 60 years old (according to the geriatric period defined by WHO) [11].

Exclusion criteria included patients with drug-induced diabetes or steroid treatment, patients receiving renal replacement therapy, patients who had undergone surgery within the three months prior to the study, patients with serious co-morbidities such as malignancy or severe infections and patients with cognitive and language impairments.

### 2.3 Data collection

Sociodemographic data were collected by trained research assistants working in the endocrinology clinics using the pretested semi-structured questionnaire. All study participants were approached during their respective appointment schedule for follow-up.

Severe hypoglycaemia (SH) was defined as the loss of consciousness and not just an event that required the assistance of someone, to avoid variable interpretation and reporting by participants. Diabetic ketoacidosis (DKA) events were those that required overnight hospitalization to ensure correct diagnosis. For both SH and DKA, we reported only the frequency of participants who experienced one or more events in the preceding 12 months, as this provided a more reliable estimate than relying solely on self-reported data.

The status of diabetes included clinic-recorded HbA1c in the last 3 months and the presence of diabetes complications assessed according to the treating physician criteria. We collected SH and DKA information both directly from the study participant and from data extraction from the medical records. Discrepancies between the two sources could be connected to under reporting in the medical records and over reporting by the study participants and their families, particularly for SH episodes that are generally not connected to hospitalization. For DKA, hospitalization at a facility was counted as an occurrence during the study period.

Additionally, blood levels of fasting glucose, glycosylated haemoglobin HbA1C and plasma levels of the inflammation markers (IL-6, P-selection and ICAM-1) were analysed by the Department of Clinical Biochemistry, Shifa Hospital. These inflammatory markers were measured to assess the potential role of systemic inflammation and endothelial dysfunction in the relationship between sleep quality and glycaemic control. Chronic inflammation and endothelial dysfunction are known contributors to insulin resistance, metabolic dysregulation and vascular complications in T2DM. Given that poor sleep quality has been associated with heightened inflammatory responses, these biomarkers were included to explore potential mechanistic links in this population. Plasma levels of inflammatory markers were measured using enzyme-linked immunosorbent assay (ELISA).

### 2.4 Study questionnaire

For our study, we developed a custom-designed questionnaire based on existing literature and the Sleep Quality questionnaire (SQQ) to assess participants' sleep quality. The questionnaire underwent a pilot study with 15 participants, recruited from diabetes clinics, to evaluate its comprehensibility and feasibility. Feedback from participants led to minor adjustments to improve clarity.

The final version of the questionnaire included 15 items, using a 3-point scale (0, 1, 2 and 3), covering five sleep health components: subjective sleep quality, sleep latency, sleep duration, use of sleep medications and daytime dysfunction. Subjective sleep quality was scored from 0 to 4, with 0 indicating very good sleep quality, 1 indicating fairly good, 2 neutral, 3 fairly bad and 4 very bad. Sleep latency was scored based on the time it took to fall asleep each night, with 0 for less than 15 min, 1 for 15–20 min, 2 for 21–30 min, 3 for 31–45 min and 4 for more than 45 min. Sleep duration was scored as follows: 0 for more than 7 h, 1 for 6 to ≤7 h, 2 for 5 to <6 h, 3 for 4 to <5 h and 4 for less than 4 h. The use of sleep medications was scored from 0 to 4, with 0 for not used during the past month, 1 for less than once a week, 2 for 1–2 times a week, 3 for 3 or more times a week and 4 for daily use. Daytime dysfunction was scored based on the frequency of difficulty staying awake, eating meals, engaging in social activities and low motivation to complete routine tasks, using the same 0 to 4 scale.

The total score ranged from 0 to 15, with higher scores indicating poorer sleep quality. A mean item score of 1.0–2.0 was considered indicative of adequate sleep quality, while a mean score greater than 3.1 was considered poor sleep quality. Participants were categorized into two groups based on their scores: those with a score of 5 or lower were classified as ‘Adequate Sleep Quality’ (ASQ), and those with a score of 10 or higher as ‘Poor Sleep Quality’ (PSQ).

The questionnaire was translated into Urdu using the back-translation technique by a proficient translator. An independent translator, blinded to the original version, translated it back into English and a consensus was reached. The Urdu version demonstrated strong internal consistency with a Cronbach's Alpha (α) of 0.85 and a reliability coefficient of 0.82. The final questionnaire was administered to all study participants by research assistants during their clinic visit.

The questionnaires were completed either through self-administration or by interview for participants who were illiterate. To minimize bias, the researchers responsible for analysing the results were kept blinded.

### 2.5 Statistical analysis

All statistical analyses was performed using IBM SPSS version 25.0. Descriptive statistics were used for the demographic characteristics of the study participants. Categorical variables were summarized as counts (percentages), while continuous variables were expressed as means (standard deviations, denoted as SD). Categorical outcome measures were analysed with the chi-square test, while continuous measures were assessed using the *t*-test or the Wilcoxon rank-sum test, as appropriate. To investigate the relationships between HbA1c levels and occurrences of hypoglycaemic or hyperglycaemic episodes, correlation analysis was conducted. Univariate and multivariate linear regression analyses were performed to assess the relation between the components of the sleep scores and HbA1c or between sleep duration and HbA1c after adjustment for confounders. Statistical significance was established with a two-tailed P value threshold of <0.05.

Based on the literature, the geometric standard deviation (SD) of change in HbA1c at the last observation period was assumed to be 0.7^13^. We determined that a minimum of 200 participants were required to achieve a statistical power of 0.80, assuming a significance level of 0.05 and an effect size of 0.3 and accounting for potential dropouts from the study. This sample size was calculated using G∗power, considering the expected variability within the population.

## 3 Results

**Fig. 2:**
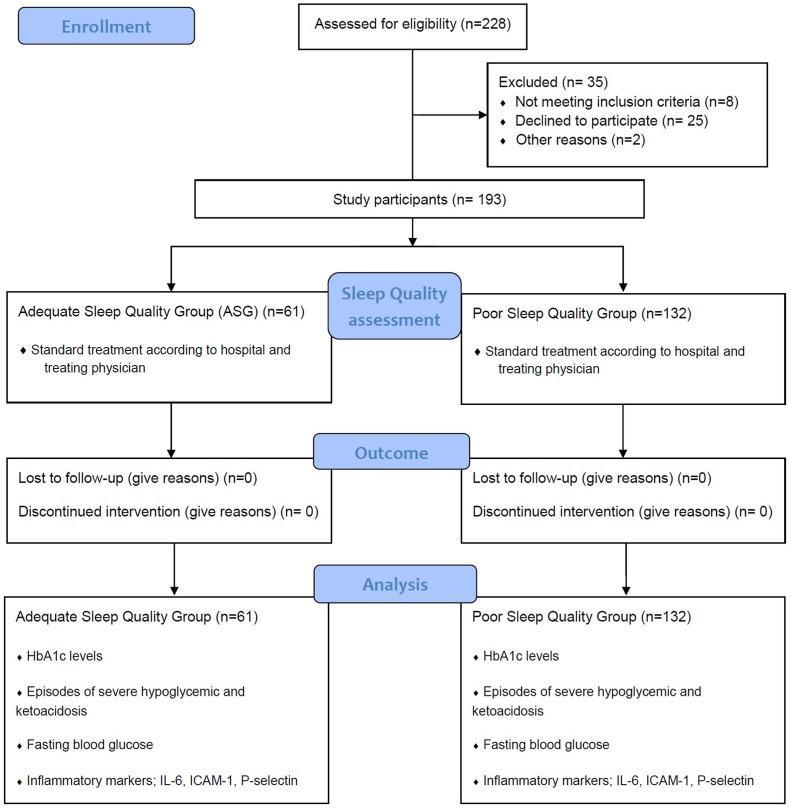
STROBE flow diagram. After 228 T2DM geriatric patients were screened for eligibility, 57 were excluded and 193 eligible participants were enrolled in the study, underwent sleep quality assessment and were classified into Poor Sleep Quality (PSQ) or Adequate Sleep Quality (ASQ) groups. Outcome measures, including fasting blood glucose, HbA1c measurements and hospital admissions related to hypoglycaemic or hyperglycaemic episodes, were recorded. Data from both PSQ and ASQ groups were analysed to evaluate the impact of sleep quality on glycaemic control.

A total of 228 patients were identified in which 25 patients declined to participate. By the time of the study commencement, 10 patients who were initially recruited withdrew for reasons unrelated to the study, including time constraints, missed appointments, divorce and a loss of interest due to the lack of financial incentives. As a result, 193 participants completed the study and provided all the necessary data. Fig. 2 outlines the flow of participants through each phase of the study.

### 3.1 Patient demographics

The study population consisted mainly of females, with a proportion of 52.3 % (101/193). The mean age of the patients was 65.2 (1.9) years. Differences in baseline information were not statistically significant between the two groups (p > 0.05.). Table 1 shows the study population characteristics.

**Table 1:** Population characteristics and differences between patients in ASQ group (n = 132) and PSQ group (n = 61). The table includes results from chi-square tests comparing both groups in terms of education, annual household income, duration of diabetes, age at diagnosis, HbA1c values and the number of severe hypoglycaemic and ketoacidotic episodes in the 12 months prior to the study.

Variable	Combined population (n = 193) (mean ± SD)	ASG group (n = 61) (mean ± SD)	PSG group (n = 132) (mean ± SD)	p value (ASG vs PSG)
**Age**	65.2 ± 1.9	64.3 ± 1.1	66.1 ± 2.8	0.76
**Education Achieved**				
No formal education	107 ± 1.7	35 ± 1.8	72 ± 1.6	0.10
First to eleventh grade	30 ± 2.3	10 ± 1.6	20 ± 2.9	0.38
High school graduate	35 ± 1.9	21 ± 1.2	14 ± 2.7	0.87
College graduate	21 ± 2.2	10 ± 3.4	11 ± 1.1	0.24
**Household annual income ($)**				
≤ $5000	101 ± 4.9	31 ± 5.6	70 ± 4.2	0.44
$5001 – $10,000	38 ± 2.3	19 ± 3.6	19 ± 1.0	0.38
$10,001-$20,000	29 ± 1.4	15 ± 1.7	14 ± 1.1	0.78
Not aware of income	25 ± 1.7	13 ± 1.8	12 ± 1.6	0.91
**Duration of diabetes (years)**	1.5 ± 1.6	1.4 ± 1.9	1.5 ± 1.3	0.79
**Age at diagnosis (years)**	62.9 ± 1.3	61.8 ± 1.2	62.3 ± 1.4	0.78
**HbA1c values (%)**	7.9 ± 1.5	7.2 ± 1.2	8.7 ± 1.9	**0.03**
**Number of ketoacidosis episodes (previous 12 months)**	77 ± 4.1	5 ± 1.7	72 ± 3.4	**0.01**
**Number of severe hypoglycaemic episodes (previous 12 months)**	43 ± 2.9	8 ± 2.1	35 ± 1.4	**0.02**
**IL-6 (ng/L)**		82.5 ± 6.2	51.8 ± 3.2	**<0.01**
**ICAM-1 (ng/L)**		434.6 ± 32.0	689.1 ± 51.8	**0.001**
**P-selectin (ng/L)**		181.1 ± 64.0	262.1 ± 81.0	**< 0.001**

ASQ: Adequate sleep quality, PSG: Poor sleep quality, HbA1c: glycosylated haemoglobin.

### 3.2 Characteristics of sleep

The prevalence of sleep issues was notably higher in PSG group, constituting 132/193 (68.7 %) patients. Different aspects of sleep health were as follows.

#### 3.2.1 Subjective sleep quality

In PSG group, subjective sleep quality was notably compromised, with a significantly higher score (51.4 ± 6.2 vs 31.7 ± 4.5) compared to ASG group. Responses indicated that PSG group described their sleep as restless, unsatisfactory and disrupted.

#### 3.2.2 Sleep latency

PSG group experienced prolonged sleep latency, with an average time to fall asleep of 35 ± 9.2 min, compared to Group ASG where mean sleep latency was notably shorter at 15 ± 6.4 min. This suggests that there was a significant delay in falling asleep among those with poor sleep quality.

#### 3.2.3 Sleep duration

PSG group exhibited significantly shorter sleep duration, averaging 5 h and 42 ± 0:48 min, while ASG group achieved an average sleep duration of 7 h and 18 ± 0:56 min. These results highlight a substantial difference in sleep duration between the two groups.

#### 3.2.4 Use of sleep medications

PSG group reported a significantly higher reliance on sleep medications, with 72 % of participants indicating their use compared to only 22 % in ASG group who resorted to sleep medications. This disparity emphasizes the severity of sleep-related issues in the PSG group. However, among those that were resorting to using sleep medications, only 15 % were using prescribed medications while 75 % were using non-prescribed sleep medications in PSG group. This was similar to ASG group with those who were using sleep medications were predominantly (85 %) using non prescribed ones and a smaller proportion were on prescription medications (15 %).

#### 3.2.5 Daytime dysfunction

Daytime dysfunction was more prevalent in PSG group, with 82 % of participants reporting significant daytime impairments. This was indicated by higher scores for symptoms such as excessive sleepiness, fatigue and difficulty concentrating. In contrast, ASG group had a substantially lower prevalence of daytime dysfunction, with only 18 % experiencing significant daytime impairments.

### 3.3 Glycaemic control, severe hypoglycaemic and ketoacidotic episodes

PSQ group exhibited higher HbA1c levels (8.7 ± 1.9 vs. 7.2 ± 1.2) compared to ASQ group. The significant difference in HbA1c values was found between the two groups was 1.5 % (p < 0.01), indicating poorer glycaemic control in the PSQ group.

The number of severe hypoglycaemic and ketoacidotic or hyperglycemic episodes were statistically significant between both groups. PSQ group had a higher number of severe hypoglycaemic (35 ± 1.4 vs. 8 ± 2.1; p 0.02), and ketoacidotic episodes (72 ± 1.0 vs. 5 ± 1.1; p = 0.01) in 12 months when compared to the ASQ group (Table 1).

### 3.4 Inflammatory markers

PSG group had higher plasma levels of IL-6, ICAM-1 and P-selectin compared to ASG group (p < 0.01), indicating a more pronounced inflammatory and endothelial activation response in the PSG group.

### 3.5 Correlation between HbA1c% and number of hypoglycaemic and ketoacidotic episodes

**Fig. 3:**
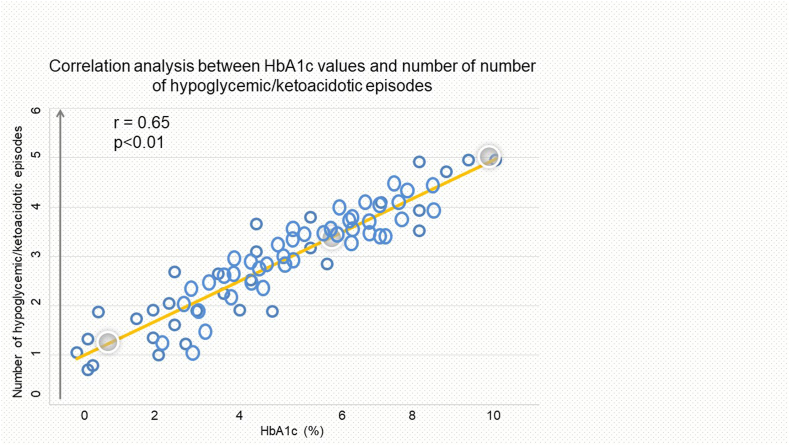
Linear association between glycosylated haemoglobin A1c (HbA1c%) and number of hypoglycaemic and ketoacidotic episodes.

A significant linear correlation was observed between lower HbA1c levels and reduced number of severe hypoglycaemic and ketoacidotic episodes during the 12 months leading up to the end of the study. Participants with higher HbA1c levels, predominantly from the PSQ group, experienced a higher frequency of these episodes, with a statistically significant difference observed between the groups. The linear relationship between HbA1c levels and the number of hypoglycaemic and ketoacidotic episodes (r = 0.65, p < 0.01) (Fig. 3).

## 4 Discussion

Newly diagnosed T2DM geriatric patients require regular support from their healthcare providers to navigate diabetes management. Glycemic control, particularly HbA1c, remains central to diabetes care, as chronic hyperglycaemia is associated with long-term organ damage [12]. Diabetes management in older adults is particularly complex due to age-related physiological changes, multiple morbidities and the need for individualized treatment approaches [12]. Understanding how sleep quality influences glycaemic control in this population is essential for optimizing care strategies.

Sleep quality significantly affects glycaemic control in T2DM, yet it remains an often overlooked factor in clinical practice. Studies indicate that a large proportion of T2DM patients experience poor sleep quality, leading to elevated glucose levels and greater variability in glycaemic control [13,14]. Our study uniquely focuses on newly diagnosed geriatric T2DM patients in South Asia, providing critical insights into sleep disturbances, diabetes management and regional healthcare challenges. Notably, the majority of our participants reported poor sleep quality, emphasizing the importance of addressing sleep in early diabetes care. Sleep disturbances in this population may be attributed to multiple factors, including physiological changes associated with aging, diabetes-related complications, polypharmacy and psychosocial stressors related to a new diagnosis. Moreover, cultural and socioeconomic factors in South Asia may further influence sleep patterns, with disparities in healthcare access, dietary habits and stress management playing significant roles.

Our study revealed significant differences between patients with poor sleep quality (PSG) and those with adequate sleep quality (ASG). PSG individuals experienced more pronounced sleep disturbances, longer sleep latency and shorter sleep durations. These findings align with previous research linking inadequate sleep with insulin resistance and systemic inflammation [[15], [16], [17]]. Sleep deprivation has been shown to contribute to sympathetic nervous system over activation, increased cortisol secretion and dysregulation of glucose metabolism, all of which can lead to poor glycaemic control [18,19]. Our results suggest that sleep disturbances in newly diagnosed T2DM may stem from acute metabolic dysregulation and the psychological stress of diagnosis rather than chronic conditions alone. The increased stress associated with a new diabetes diagnosis may contribute to difficulties in initiating and maintaining sleep, exacerbating the bidirectional relationship between poor sleep and diabetes progression [20,21].

A key finding was the significantly higher HbA1c levels in PSG compared to ASG, highlighting the link between sleep quality and glycaemic control. Additionally, PSG participants reported a higher frequency of nocturnal hypoglycaemia and increased inflammatory markers (IL-6, ICAM-1 and P-selectin), reinforcing the role of sleep disturbances in metabolic dysregulation. Nocturnal hypoglycaemia may disrupt sleep architecture by triggering autonomic responses such as night sweats, palpitations and awakenings, further exacerbating sleep fragmentation [22]. Conversely, poor sleep quality itself may increase the risk of hypoglycaemic episodes by impairing glucose counter regulation and increasing insulin resistance [23]. This bidirectional relationship suggests that interventions aimed at improving sleep quality could have meaningful effects on glycaemic stability in older adults with newly diagnosed T2DM. Future research should investigate whether improved sleep interventions can lead to long-term stabilization of blood glucose levels and reduce diabetes-related complications.

The high prevalence of sleep medication use suggests a need for better non-pharmacological sleep interventions in diabetes care. While sleep medications may provide short-term relief, their long-term use is associated with adverse effects, including cognitive impairment, falls and dependency, particularly in the elderly [24]. Our findings highlight the importance of incorporating behavioural and cognitive interventions, such as cognitive-behavioural therapy for insomnia (CBT-I), relaxation techniques and sleep hygiene education, into diabetes care plans. Future research should explore the effectiveness of such interventions in improving both sleep quality and metabolic outcomes in this population. Furthermore, developing culturally appropriate sleep intervention programs tailored to older adults in low-income settings could significantly improve adherence and patient outcomes.

Poor sleep quality also contributed to greater daytime dysfunction, with the majority of PSG participants reporting excessive sleepiness, fatigue and difficulty concentrating. These impairments can exacerbate diabetes self-management challenges, further emphasizing the necessity of integrating sleep assessments into routine diabetes care. Cognitive impairment and reduced executive function due to sleep disturbances can negatively affect medication adherence, dietary choices and physical activity levels, ultimately leading to suboptimal glycaemic control [25]. Addressing sleep issues early in diabetes management may enhance patient engagement in self-care behaviours and improve long-term health outcomes. Additionally, healthcare professionals should receive training on the identification and management of sleep disturbances in geriatric diabetes care to ensure that these factors are routinely considered in treatment plans.

Our study's novelty lies in its focus on newly diagnosed T2DM geriatric patients, an understudied population, particularly in low-income settings like Pakistan. The intersection of sleep disturbances, diabetes management and regional healthcare disparities provides new perspectives on improving care for this vulnerable group. By establishing the significant association between sleep quality and glycaemic control in this group, our findings underscore the need for early interventions to optimize sleep hygiene and improve diabetes outcomes. Given the growing prevalence of diabetes among older adults, understanding these relationships is crucial for designing targeted interventions that enhance both metabolic health and quality of life. Additionally, these findings add to the growing body of evidence emphasizing the importance of sleep as a modifiable risk factor in chronic disease management.

The study has several limitations. First, sleep data were recorded only at baseline, preventing us from tracking changes over time. A single measure may not fully capture sleep quality's long-term effects on glycaemic control in T2DM. Additionally, other variables may have changed during follow-up, but we could not account for them. Second, analysis relied on self-reported sleep data, which are less reliable than objective measures like polysomnography, which were not used due to cost. However, research shows subjective sleep reports moderately correlate with objectives measures and self-reported data have been effectively used in similar studies. Furthermore, certain aspects of sleep quality, such as sleep segments, were not assessed. Third, selection bias is possible, as 25 patients declined participation and 10 withdrew before the study began. Nonparticipants may have had different sleep or glycaemic profiles, potentially affecting generalizability. Future studies with larger, more diverse samples are needed for validation. Fourth, the 15-item sleep questionnaire was based on existing literature and the Sleep Quality Questionnaire (SQQ) but lacks full psychometric validation. A pilot study ensured clarity and feasibility, yet further validation against standardized measures is required. Finally, the sample comprised geriatric South Asian individuals in a healthcare setting, limiting generalizability to other populations and settings.

It is important to note that this study focused on newly diagnosed T2DM patients, which may limit the applicability of our findings to individuals with long-standing diabetes. Chronic hyperglycaemia, diabetes-related complications and prolonged use of medications may further influence sleep parameters. Patients with well-controlled long-standing T2DM may experience fewer sleep disturbances, whereas those with poorly controlled diabetes may exhibit more severe sleep-related issues due to complications such as neuropathy or nocturnal hypoglycaemia. Future studies comparing newly diagnosed versus long-standing T2DM patients could provide further insights into these relationships.

## 5 Conclusion

In conclusion, this study underscores the substantial impact of habitual sleep quality on glycaemic control in geriatric patients with T2DM. The results suggest that poor sleep quality is associated with compromised glycaemic control and an increased risk of severe hypoglycaemic and ketoacidotic episodes. Healthcare providers and diabetes management teams should consider routine assessments of sleep quality in their treatment plans for older adults with diabetes. Addressing sleep disturbances could improve glycaemic control but also improve the overall quality of life for this vulnerable population. Future research should focus on developing targeted interventions to address sleep issues as a modifiable factor in diabetes management strategies.

## CRediT authorship contribution statement

**Nadia Hussain:** Writing – original draft, Supervision, Data curation, Conceptualization. **Amal Hussain Ibrahim Al Haddad:** Writing – original draft, Methodology, Formal analysis. **Saima Abbass:** Writing – original draft, Supervision, Data curation, Conceptualization. **Zina Alfahl:** Writing – review & editing, Writing – original draft, Conceptualization.

## Data availability

Data can be made available upon reasonable request.

## Declaration of generative AI

AI and AI-assisted technologies were not used in the writing process for this manuscript.

## Funding

The study was funded by the Shifa Hospital Research Council grant [SIAC-02521]. The funders did not have any role in the study design, execution, data collection or analysis and in the manuscript preparation or editing. Study ID: UMIN ID: UMIN000051181.

## Declaration of competing interest

The authors declare that they have no known competing financial interests or personal relationships that could have appeared to influence the work reported in this paper.

## References

[1] Das U., Kar N. (Aug 11 2023). Prevalence and risk factor of diabetes among the elderly people in West Bengal: evidence-based LASI 1st wave. BMC Endocr Disord.

[2] Saeedi P., Petersohn I., Salpea P. (Nov 2019). Global and regional diabetes prevalence estimates for 2019 and projections for 2030 and 2045: results from the International Diabetes Federation Diabetes Atlas, 9(th) edition. Diabetes Res Clin Pract.

[3] Azeem S., Khan U., Liaquat A. (Jul 2022). The increasing rate of diabetes in Pakistan: a silent killer. Ann Med Surg (Lond)..

[4] Zheng Y., Ley S.H., Hu F.B. (Feb 2018). Global aetiology and epidemiology of type 2 diabetes mellitus and its complications. Nat Rev Endocrinol.

[5] Yu M., Zhan X., Yang Z., Huang Y. (Aug 2021). Measuring the global, regional, and national burden of type 2 diabetes and the attributable risk factors in all 194 countries. J Diabetes.

[6] Sakamoto R., Yamakawa T., Takahashi K. (2018). Association of usual sleep quality and glycemic control in type 2 diabetes in Japanese: a cross sectional study. Sleep and Food Registry in Kanagawa (SOREKA). PLoS One.

[7] Zhu B., Vincent C., Kapella M.C. (Jan 2018). Sleep disturbance in people with diabetes: a concept analysis. J Clin Nurs.

[8] Rutters F., Nefs G. (2022). Sleep and circadian rhythm disturbances in diabetes: a narrative review. Diabetes Metab Syndr Obes.

[9] Jang J.H., Kim W., Moon J.S. (Apr 16 2023). Association between sleep duration and incident diabetes mellitus in healthy subjects: a 14-year longitudinal cohort study. J Clin Med.

[10] ElSayed N.A., Aleppo G., Aroda V.R. (2023). Summary of revisions: standards of care in diabetes—2023. Diabetes Care.

[11] (2020). World health O. Ageing.

[12] Ashrafzadeh S., Hamdy O. (Mar 5 2019). Patient-driven diabetes care of the future in the technology era. Cell Metab.

[13] Darraj A. (Nov 2023). The link between sleeping and type 2 diabetes: a systematic review. Cureus.

[14] Chattu V.K., Manzar M.D., Kumary S., Burman D., Spence D.W., Pandi-Perumal S.R. (Dec 20 2018). The global problem of insufficient sleep and its serious public health implications. Health Care.

[15] Denison H.J., Jameson K.A., Sayer A.A. (Apr 2021). Poor sleep quality and physical performance in older adults. Sleep Health.

[16] Hur M.H., Lee M.K., Seong K., Hong J.H. (Oct 2020). Deterioration of sleep quality according to glycemic status. Diabetes Metab J.

[17] Sansom K., Reynolds A., McVeigh J. (2023). Estimating sleep duration: performance of open-source processing of actigraphy compared to in-laboratory polysomnography in the community. Sleep Adv.

[18] Schipper S.B.J., Van Veen M.M., Elders P.J.M. (Nov 2021). Sleep disorders in people with type 2 diabetes and associated health outcomes: a review of the literature. Diabetologia.

[19] Shan Z., Ma H., Xie M. (Mar 2015). Sleep duration and risk of type 2 diabetes: a meta-analysis of prospective studies. Diabetes Care.

[20] Reutrakul S., Van Cauter E. (Jul 2018). Sleep influences on obesity, insulin resistance, and risk of type 2 diabetes. Metabolism.

[21] Lee D.Y., Jung I., Park S.Y. (Feb 2023). Sleep duration and the risk of type 2 diabetes: a community-based cohort study with a 16-year follow-up. Endocrinol Metab (Seoul).

[22] Ogilvie R.P., Patel S.R. (Aug 17 2018). The epidemiology of sleep and diabetes. Curr Diabetes Rep.

[23] Engert L.C., Mullington J.M., Haack M. (Oct 2023). Prolonged experimental sleep disturbance affects the inflammatory resolution pathways in healthy humans. Brain Behav Immun.

[24] Bhati P., Hussain M.E. (Oct 2019). Sleep duration is a significant predictor of cardiac autonomic neuropathy in type 2 diabetes mellitus. Prim Care Diabetes.

[25] Garg H. (Sep-Oct 2018). Role of optimum diagnosis and treatment of insomnia in patients with hypertension and diabetes: a review. J Fam Med Prim Care.

